# 90 Outcomes of Minimally Invasive Excision with Epidermal Autografting in Pediatric Partial Thickness Burns

**DOI:** 10.1093/jbcr/irae036.089

**Published:** 2024-04-17

**Authors:** Julia Maxey, Mallory Wampler, Djoni Elkady, Kelly Williamson, Richard B Lou, Anjay Khandelwal

**Affiliations:** Cleveland Clinic, Ghent, Ohio; Akron Children's Hospital, Stow, Ohio; Loyola University Chicago Stritch School of Medicine, North Olmsted, Ohio; Akron Children's Hospital, Akron, Ohio; Cleveland Clinic, Ghent, Ohio; Akron Children's Hospital, Stow, Ohio; Loyola University Chicago Stritch School of Medicine, North Olmsted, Ohio; Akron Children's Hospital, Akron, Ohio; Cleveland Clinic, Ghent, Ohio; Akron Children's Hospital, Stow, Ohio; Loyola University Chicago Stritch School of Medicine, North Olmsted, Ohio; Akron Children's Hospital, Akron, Ohio; Cleveland Clinic, Ghent, Ohio; Akron Children's Hospital, Stow, Ohio; Loyola University Chicago Stritch School of Medicine, North Olmsted, Ohio; Akron Children's Hospital, Akron, Ohio; Cleveland Clinic, Ghent, Ohio; Akron Children's Hospital, Stow, Ohio; Loyola University Chicago Stritch School of Medicine, North Olmsted, Ohio; Akron Children's Hospital, Akron, Ohio; Cleveland Clinic, Ghent, Ohio; Akron Children's Hospital, Stow, Ohio; Loyola University Chicago Stritch School of Medicine, North Olmsted, Ohio; Akron Children's Hospital, Akron, Ohio

## Abstract

**Introduction:**

The traditional approach to pediatric deep partial thickness burns has been a “watch and wait” attitude with frequent dressing changes, primarily due to evidence that pediatric burns will often heal, and that early debridement leads to removal of non-viable tissue. However, there is still significant morbidity with delayed healing, increased pain and dressing changes, prolonged hospital stays, added cost and hypertrophic scarring. Dermabrasion is a minimally invasive excisional technique that may preserve viable dermis while epidermal autografting can be used for partial thickness burns to facilitate wound healing. In an effort to improve outcomes, the authors evaluated the outcomes of dermabrasion with epidermal autografting in the pediatric population.

**Methods:**

A retrospective review of pediatric patients (< 18 years old) who underwent minimally invasive excision using dermabrasion with epidermal autografting as the only primary surgical intervention between January 2022 and July 2023 was performed. Patient information collected included: demographics, burn depth, mechanism of injury, percentage of total burn surface area (%TBSA), time to operating room (OR), length of stay (LOS), narcotic use (morphine equivalents), postoperative complications, need for autografting, and number of dressing changes requiring sedation. This was compared to previously conservatively managed patients as well as published historical data.

**Results:**

A total of 46 patients [mean age: 4.95 years (range: 0.04-18)] were examined with an average %TBSA of 7.45 (range: 0.3-19.75). The majority of patients sustained a scald injury (74%). Most patients (66%) had involvement of critical areas including the hands, face, feet or genitalia. Approximately 40% of the patients had Fitzpatrick Skin Type V-VI. The average time to OR was 2.7 days (range 0-8). Average length of stay/%TBSA was 0.45 days. The total number of dressing changings requiring sedation was 1.6 (range 0-7). Almost all patients (96%) had wounds that were >90% re-epithelialized by postoperative day 10. When compared to historical data, time to epithelialization was decreased by 5 days, grafting rate was decreased (11.5% to 6%), opiate usage was decreased in half (0.3 MME/kg/day to 0.15 MME/kg/day), infection rate was reduced to zero, and average LOS was decreased from 10.4 days to 3.6 days).

**Conclusions:**

Within the pediatric population, minimally invasive excision with epidermal autografting may be superior to conservative management with decreased length of stay, fewer dressing changes, decreased infections, decreased need for autografting and decreased opiate consumption. Length of stay can be significantly decreased with earlier operating room availability and swift decision making.

**Applicability of Research to Practice:**

There may be a viable alternative option to the traditional “watch and wait” approach to pediatric partial thickness burns.

